# Asthma-COPD Overlap in Clinical Practice (ACO_CP 2023): Toward Precision Medicine

**DOI:** 10.3390/jpm13040677

**Published:** 2023-04-18

**Authors:** Ahmad R. Alsayed, Mahmoud S. Abu-Samak, Mohammad Alkhatib

**Affiliations:** 1 Department of Clinical Pharmacy and Therapeutics, Faculty of Pharmacy, Applied Science Private University, Amman 11937, Jordan; 2 Department of Experimental Medicine, University of Rome “Tor Vergata”, 00133 Roma, Italy; mohammad--alkhatib@hotmail.com

**Keywords:** ACO, asthma, COPD, phenotype, clinical guide, personalized medicine, precision medicine

## Abstract

Asthma and COPD have characteristic symptoms, yet patients with both are prevalent. Despite this, there is currently no globally accepted definition for the overlap between asthma and COPD, commonly referred to as asthma–COPD overlap (ACO). Generally, ACO is not considered a distinct disease or symptom from either clinical or mechanistic perspectives. However, identifying patients who present with both conditions is crucial for guiding clinical therapy. Similar to asthma and COPD, ACO patients are heterogeneous and presumably have multiple underlying disease processes. The variability of ACO patients led to the establishment of multiple definitions describing the condition’s essential clinical, physiological, and molecular characteristics. ACO comprises numerous phenotypes, which affects the optimal medication choice and can serve as a predictor of disease prognosis. Various phenotypes of ACO have been suggested based on host factors including but not limited to demographics, symptoms, spirometric findings, smoking history, and underlying airway inflammation. This review provides a comprehensive clinical guide for ACO patients to be used in clinical practice based on the available limited data. Future longitudinal studies must evaluate the stability of ACO phenotypes over time and explore their predictive powers to facilitate a more precise and effective management approach.

## 1. Introduction

Asthma and chronic obstructive pulmonary disease (COPD) have unique clinical features. Nevertheless, these clinical features can coexist, making it difficult to distinguish between the two conditions in a clinical setting. As a result, the term “asthma–COPD overlap” (ACO) was introduced to describe a group of clinical characteristics rather than a single entity [1,2]. Based on clinical and molecular perspectives, ACO is unlikely to be a single disease or syndrome, but rather a complex interaction and combination of different pathophysiological mechanisms. Identifying patients with ACO is crucial to guiding clinical care. Given the multiple phenotypes associated with ACO, it is challenging to create a single definition. Various societies and researchers have attempted to define ACO by utilizing clinical characteristics [3,4], spirometry [5], or both [6]. Nonetheless, there is currently no globally accepted definition of ACO. 

Most proposed definitions of ACO require the patient to be over 40, have airflow limitation, and have asthma or partial bronchodilator (BD) reversibility [7]. The term ACO is favored over ACOS (ACO syndrome) since there is no singular disease or syndrome.

In 2015, the Global Initiative for Asthma (GINA) and the Global Initiative for Chronic Obstructive Lung Disease (GOLD) issued a collaborative statement defining what was formerly known as ACOS. They defined ACOS as the presence of persistent airflow limitation with characteristics commonly associated with both asthma and COPD [8]. Hence, in clinical practice, ACOS is identified by the overlapping features it shares with both asthma and COPD.

Then in 2017, the American Thoracic Society (ATS) and the National Heart, Lung, and Blood Institute (NHLBI) collaborated on an ACO workshop report. The panel concluded that ACO, like asthma and COPD, does not represent a singular unique disease entity [1]. Instead, ACO should be utilized to describe patients with a prolonged history of asthma, a modest smoking history, and fixed-airflow obstruction. Concurrently, those who exhibit asthma-like symptoms, such as peripheral eosinophilia and bronchodilator responsiveness, may have ACO. While both patient groups are clinically and likely pathologically unique, they are both within the purview of ACO.

Other entities and groups have suggested further major and minor characteristics that may aid in identifying patients with ACO, as detailed in Table 1 [8,9,10,11,12,13,14]. At the same time, the 2020 GOLD Strategy update dismissed the term “asthma–COPD overlap” because asthma and COPD are distinct illnesses that may have overlapping characteristics, such as eosinophilia or a degree of reversibility [3]. Furthermore, GOLD indorses that concurrent diagnoses of asthma and COPD may happen in a single patient, and in such cases, that treatment should generally adhere to asthma guidelines. However, COPD-specific therapeutic methods may be necessary for some individuals.

This review provides a comprehensive clinical guide for ACO patients to be used in clinical practice based on the available limited data. This review covers an overview of the epidemiology, pathogenesis, and diagnosis, followed by non-pharmacological and pharmacological treatment options and future directions.

## 2. Epidemiology

The lack of a standardized definition for ACO makes it challenging to estimate its disease burden accurately. According to self-reported physician diagnosis or a combination of both spirometry and symptom reporting, the prevalence of ACO in the overall population ranges from 2 to 3 percent, whereas the prevalence of asthma and COPD ranges from 5 to 17 percent and 2 to 12 percent, respectively [7]. However, in COPD patients, the prevalence of ACO may be as high as 25 percent [15], and in asthma patients, the estimated prevalence of ACO ranges from 10 to 31 percent [7]. 

Studies indicate that individuals with ACO are more likely to be female, have a higher body mass index, and possess lower education and socioeconomic levels than those with COPD. However, there is considerable variety within this patient population [16,17,18,19]. The data concerning the outcomes of patients with ACO compared to those with either asthma or COPD without overlap are varied, possibly due to the heterogeneity of patients included in the ACO umbrella term. For example, such variation can be observed in a population-based cohort study that followed patients for a median of nine years. In this study, patients with ACO and patients with COPD had comparable higher risks of exacerbations and all-cause mortality than those of symptomatic smokers without COPD [20]. In contrast, other studies suggest that disease control in ACO individuals may be worse than in those with asthma or COPD alone, especially regarding lung function, exacerbation rates, and symptoms [21]. Furthermore, patients with ACO have a similar risk of developing lung cancer as those with COPD, and a higher risk than other groups of smokers [22].

## 3. Pathogenesis

Whether ACO is caused by a unique pathogenic mechanism or by asthma and COPD coexisting in the same patient is a common question. Certainly, even the pathophysiology of COPD is contested. Likewise, the pathogenic processes that underlie asthma are diverse. 

The Dutch hypothesis proposes that asthma and COPD are caused by a single disease entity, however the clinical phenotype is influenced by various factors such as genetics and environmental exposures [23,24]. Instead, the British hypothesis proposes that asthma and COPD have independent origins, each with its own characteristic inflammatory causes, such as allergic inflammation in asthma and persistent bacterial infection in COPD [25]. According to the Dutch hypothesis, ACO would fall on the same continuous spectrum as asthma and COPD. However, according to the British hypothesis, different causes would drive distinctive inflammation, distinguishing ACO from asthma and COPD.

Hypothetically, it is easy to recognize how smoking tobacco can increase neutrophilic inflammation, cause fixed-airflow limitation, and ultimately lead to COPD in patients with asthma [26]. Conversely, a patient without asthma who is atopic “sensitive to allergens” may initially develop COPD, but eventually exhibit airway hyperresponsiveness and type 2-mediated airway inflammation. Allergen sensitivity has also been reported in older patients with COPD [27]. Given the variability in the clinical characteristics of ACO patients, it is likely that the primary inflammatory pathway differs among individuals.

The risk factors that cause ACO are crucial. Smoking, age, and airway inflammation are risk factors for greater lung function declines and permanent airway obstruction in asthma patients later in life. Similarly, COPD patients may exhibit eosinophilia or elevated BD reactivity. 

The interaction between an organism’s genome and its environment produces its phenotype [28,29]. Phenotypes are categorized based on factors such as the age of onset, triggers, and therapeutic or inflammatory responses such as eosinophilic or neutrophilic [29]. However, phenotypes alone are insufficient for identifying the underlying pathway of diseases. In addition, disease mechanisms that are functional or pathobiological are referred to as endotypes [28,29]. Molecular phenotyping can help identify causal pathways and improve patient outcomes [28]. Ultimately, endotypes are established using molecular phenotyping.

## 4. Respiratory Microbiome and ACO during Stable and Exacerbation States

Different respiratory pathogens play essential roles in the exacerbations of asthma and COPD [30,31,32,33]. ACO patients’ outcomes differ from those of asthma or COPD patients without overlap, possibly due to this group’s wide diversity of individuals. ACO patients are often excluded from research studies for asthma and COPD. Consequently, large-scale trials that analyze critical patient outcomes in well-defined ACO populations are required. The respiratory microbiome could be a promising biomarker in ACO, facilitating personalized medicine implementation.

In 2023, we published the first investigation in the literature which characterized ACO patients’ bacterial respiratory microbiome in both stable and exacerbation states [34]. The study utilized Next Generation Sequencing (NGS) to reveal the composition of the pulmonary microbiome and detect bacteria that may have been missed by traditional antimicrobial therapy due to cultural challenges. In this study, the lower airways of clinically stable ACO patients were found to have bacterial colonization, which makes it challenging to establish the role of bacteria in exacerbation. The examination also revealed significant differences between the study groups [34].

In the exacerbation condition, the taxonomic richness of bacteria decreased, and the evenness of microbiota increased compared to stable states in ACO patients. This shift in the airway bacterial population suggests that significant pathogens may replace most of the airway microbiome during an exacerbation, decreasing microbial richness [34]. The *Prevotella* genus was found to be substantially more abundant in exacerbation samples than in stable samples, suggesting that some species of *Prevotella* may contribute to ACO pathogenicity [34].

Moreover, high-throughput Illumina MiSeq, used in this recent work [34], revealed a large amount of bacterial variety in sputum. Similar to earlier COPD investigations utilizing culture techniques, this study found substantial abundances of the bacterial aerobic genera *Streptococcus*, *Haemophilus*, *Pseudomonas*, and *Moraxella* [35,36]. The investigation also detected high frequencies of *Prevotella*, *Veillonella*, and *Actinomyces* anaerobes, suggesting a rich spectrum of taxa. The composition of microbial communities in the airways differed from one patient to another. It is worth mentioning that the community structures specific to each patient, which showed a predominance of one or a few microbial genera, were in line with previous studies on COPD patients [32,37,38]. These studies showed that a particular signature persists throughout time.

An improved understanding of this association could lead to the development of more precise antibiotic therapies that can effectively target the increased number and activity of harmful bacteria, especially during exacerbations. This would enhance clinical effectiveness and improve patient outcomes.

The bacterial microbiome is influenced by various host and pathogen factors [32,39]. Further research is needed to understand the ACO bacterial microbiome, including its development, exacerbations, and treatments such as antibiotics and inhaled corticosteroids, which can alter bacteria in the stable airways of ACO patients. Bacteria can impact the progression of ACO disease and exacerbations. Moreover, antimicrobial intervention studies may be required if bacteria are found to cause unfavorable outcomes in stable ACO patients.

## 5. Clinical Presentation and Diagnosis

ACO is a condition characterized by substantial clinical variations amongst diverse subgroups or phenotypes [40]. Clinical manifestations that have been described for ACO are included in Table 1 and Table 2. The clinical term ACO has been employed for patients who display symptoms of both asthma and COPD. Nevertheless, a clear set of features that establishes a definitive diagnosis of ACO has not been universally agreed upon [1]. Despite the lack of a widely accepted definition, GINA acknowledges several characteristics that support an ACO diagnosis.

Additional airway illnesses, for example bronchiectasis, obliterative bronchiolitis, and central airway obstruction, are included in the differential diagnosis of ACO [2,4]. Table 3 represents the concluded proposed data collection for ACO diagnostic.

## 6. Proposed Treatment Guide for ACO

### 6.1. Non-Pharmacologic Treatment

Patients with ACO can benefit from general nonpharmacologic measures derived from effective therapies in managing asthma and/or COPD [3,4]. Table 4 shows the most appropriate options.

### 6.2. Pharmacologic Treatment

Agreement over the effective management of ACO is in its infancy, and there is limited consensus among medical professionals on the most effective approach. The initial pharmacological options indicated in the joint GINA and GOLD statement on ACO depend on the expert opinion of medical professionals [3]. Unfortunately, formal data on the management of ACO are rare, primarily because clinical studies of asthma and COPD medicines have usually excluded ACO patients specifically. Despite this, some practitioners take a similar approach to ACO as they do with asthma, which includes the use of inhaled corticosteroids (ICS) and adjusting the medication depending on patient responsiveness. The use of LABA and/or LAMA treatment is given nearly similar weight and is recommended as the starting point for maintenance treatment in COPD [3,4]. However, the findings of the Salmeterol Multicenter Asthma Research Trial (SMART) have shown that LABA monotherapy is not recommended in asthma [41]. This 28-week trial compared metered-dose inhaler (MDI) salmeterol to a placebo. In a preliminary study of 26,355 participants, respiratory and asthma-related mortality increased, particularly among African Americans, when using LABA therapy alone to maintain asthma control, raising safety concerns. Since then, the safety of asthma patients utilizing combined ICS-LABA inhalers has increased. Therefore, ICS-LABA combination inhalers are safe for ACO, unlike LABA monotherapy.

A randomized, open-label crossover study was conducted with 16 ACO patients as participants (determined as the combination of fixed-airflow obstruction with airway hyperresponsiveness by methacholine inhalation challenge). This trial found that 4 weeks of once-daily fluticasone furoate with vilanterol improved FEV_1_ more significantly than a run-in phase of twice-daily fluticasone propionate and salmeterol [42].

Despite treatment with ICS, some ACO patients may continue to experience exercise limits or frequent exacerbations. There is no evidence to guide subsequent modifications in medicine; a stepwise approach depending on symptoms, exacerbations, and therapy response is feasible, similar to the strategy employed in asthma [3,43].

For patients who experience persistent symptoms and/or exacerbations despite ICS/LABA or ICS/LAMA treatment, triple therapy (LAMA-LABA-ICS) is recommended.

In randomized, open-label crossover study with 17 ACO patients, adding umeclidinium (LAMA) to fluticasone furoate/vilanterol (ICS/LABA) resulted in a higher improvement in FEV_1_ after four weeks compared to continuing fluticasone furoate/vilanterol alone [44]. Patients with severe, persistent asthma or COPD with frequent exacerbations have had positive outcomes after undergoing triple therapy [3,43].

It is crucial to identify various phenotypes of ACO to develop comprehensive treatment approaches and apply targeted medicines for more precise management [40].

**Figure 1:**
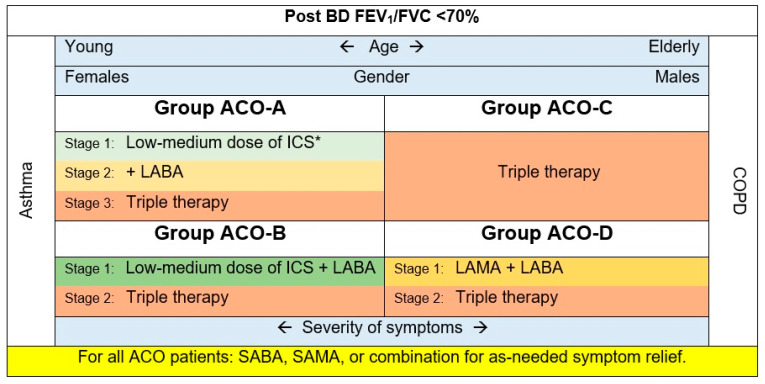
The treatment scheme for ACO patients based on the four phenotypes. * This stage can be skipped for patients with frequent exacerbations.

Based on the evidence presented in this review and previous research [5,14,40,45,46,47,48], the following recommendations are encouraged (Figure 1):

**Group ACO-A:** Asthmatics, non-smokers, most probably females. These patients may exhibit eosinophilic inflammation signs.

All patients with ACO should have easy access to a rapid onset of an inhaled BD (e.g., short-acting beta 2 agonists (SABA), a short-acting muscarinic antagonist (SAMA), or combination) for as-needed symptom relief.

Inhaled corticosteroids (ICS) are appropriate in this group of ACO, as ICS are a mainstay of asthma treatment [4]. ICS will be essential in treating patients with asthma-predominant phenotypes exhibiting eosinophilic inflammation. Following the GINA strategy, this guide suggests regular low doses of ICS. 

Adding a long-acting beta-agonist (LABA), long-acting muscarinic antagonist (LAMA), or both may be essential for symptom control. Nevertheless, as in asthma, LABA monotherapy should be avoided. Leukotriene receptor antagonists can be considered add-on treatments.

**Group ACO-B:** Asthmatics, smokers, patients having a fixed airway obstruction. These patients are typically younger females with a higher prevalence of atopic characteristics [46,47]. Their utilization of health care services is usually greater than that of groups ACO-C and D.

All ACO patients should have convenient availability to an inhaled BD with rapid onset (e.g., SABA, SAMA, or combination) for symptom alleviation as needed.

Given the limited data, we recommend starting with ICS plus LABA, then moving to the triple therapy for patients non-responsive to the first stage.

**Group ACO-C:** COPD accompanied by eosinophilia. Patients in this group tend to be older men with a higher eosinophil count than smokers (>300 cells/µL) and T helper cell type 2–related indicators [49].

All ACO patients should have convenient availability to an inhaled BD with rapid onset (e.g., SABA, SAMA, or combination) for symptom alleviation as needed.

They usually use LAMA [49]. However, accumulating evidence in COPD suggests an association between higher levels of blood eosinophils and ICS response [50,51]. In contrast, at the beginning of COPD treatment in group B COPD patients, LAMAs and LABAs are administered alone or in combination; ICS are not used as monotherapy for COPD [3]. However, the 2023 GOLD recommends starting LABA and LAMA combination for group B COPD patients and triple therapy for group E (previously known as group C and D) [52,53]. Taking these into consideration, this ACO_CP 2023 guide recommends starting with the triple therapy for this ACO phenotype.

**Group ACO-D:** COPD with a substantial BD response (FEV_1_ ≥ 15% and ≥400 mL), as defined by previous publications [14,48,49]. These patients have numerous characteristics of COPD with eosinophilia phenotype, as they are usually older males with comparable baseline lung function [49,54]. Their blood eosinophil level is typically <300 cells/µL.

All ACO patients should have convenient availability to an inhaled BD with rapid onset (e.g., SABA, SAMA, or combination) for symptom alleviation as needed.

Individuals with COPD-predominant ACO and low eosinophil levels were usually treated with LAMA. Other treatments include LABA (prescribed alone or in combination with LAMA), ICS, macrolides, and roflumilast [5]. ICS are not used as monotherapy for COPD [3]. However, in the 2023 GOLD, they recommend starting LABA and LAMA combination for group B COPD; considering that, this ACO_CP 2023 guide recommends starting with LAMA plus LABA as initial therapy for this ACO phenotype. Adding ICS (triple therapy) can be considered for unresponsive cases.

## 7. Biologic Agents

Patients who continue to have symptoms or have exacerbations despite triple therapy should be investigated for indications of one or more of the biological medicines identified for asthma. These indications include sensitivity to perennial allergens, peripheral blood eosinophilia, and/or high total serum IgE. While waiting for data in ACO patients, some clinicians often apply the same criteria as in severe persistent asthma.

Evidence on the usage of biologics in patients with ACO is infrequent and unreliable. In Australia, Omalizumab enhanced asthmatic patients’ control and quality of life [55]. Likewise, a post hoc study of PROSPERO revealed that ACO patients treated with Omalizumab had similar outcomes as asthma patients treated with the same medication [56]. Nonetheless, a study published in 2022 compared the response to biologics in asthmatics and ACO patients in the real world found that just 16% of ACO patients gained clinical control, compared to 40% of asthmatics [57]. Several monoclonal anti–IL-5 and anti–IL- 5Ra antibodies are licensed to treat eosinophilic asthma. Only mepolizumab and benralizumab have been demonstrated to reduce the rate of exacerbation in a highly chosen sample of COPD patients with elevated eosinophil levels [58]. Dupilumab, an anti–IL-4Ra antibody, has been demonstrated to enhance lung function and exacerbations in individuals with severe asthma, with a more remarkable improvement in patients with greater eosinophil levels [59]. However, its efficacy in individuals with COPD and ACO has not been confirmed. Although biologic medicines are not currently the usual care for ACO, selecting a medication with demonstrated effectiveness in asthma and eosinophilic COPD may be prudent.

Table 5 describes the limited evidence on using biological agents in COPD that may notify therapy selection in patients with ACO features.

### 7.1. Anti-IgE Therapy

Omalizumab’s forty-eight weeks observational study analyzed patients with asthma who were not excluded because they had concomitant COPD or previous smoking history [56]. Using different ACO definitions, patients in this study who met the criteria for ACO showed the same improvement in exacerbation rates and symptoms as patients who did not meet the ACO criteria, compared to rates before omalizumab use.

The Australian Xolair Registry found that patients with a COPD diagnosis based on physician assessment or fixed-airflow obstruction had better asthma control and health-related quality of life ratings but no significant change in FEV_1_ [62].

A case series of ten ACO patients found that the IL-4 levels were lower and respiratory symptoms were improved [63]. In small trials such as this, it is a challenge to ascertain whether the patients investigated had ACO or severe asthma that was not reversible post-BD. 

### 7.2. Anti-IL-5/IL-5 Receptor Alpha (IL-5Ra) Therapies

In eosinophil development, maturation, and tissue migration, IL-5 is a crucial mediator. Several anti-IL-5 and anti-IL-5Ra monoclonal antibodies are licensed as maintenance treatments for severe asthma and eosinophilia [66,67,68].

While previous phase 2 research with benralizumab indicated a trend toward exacerbation reductions in COPD patients with blood eosinophil concentrations of ≥200 cells/µL [72], two phase 3 COPD research findings were undesirable. Mepolizumab outcomes for COPD are similarly variable. A phase 3 study of COPD patients with blood eosinophil levels of 150 cells/µL at screening or 300 cells/L the year prior revealed a clinically and statistically significant decrease in mild or severe exacerbations. The parallel investigation was unsuccessful [73]. Consequently, additional evidence will be required to determine whether ACO patients may benefit.

### 7.3. IL-4 Receptor Alpha (IL-4Ra) and IL-13 Therapies 

IL-4 and IL-13 contribute to allergic inflammation by attracting eosinophils and mast cells to locations of allergic inflammation and inducing goblet cell metaplasia [74]. 

Lebrikizumab and tralokinab, two anti-IL-13 antibodies, failed to prevent asthma exacerbations in phase 3 trials and are not now licensed for clinical usage [75,76]. 

## 8. Future Directions

To better understand ACO and its treatment, further investigation is needed to identify the types of biomarkers and phenotypes that can identify patients who are most sensitive to particular therapies. For instance, in certain patients, incompatibility of illness course and clinical presentation with some pulmonary diseases such as asthma or COPD results in the patient’s status becoming more complicated in the face of optimal therapeutic protocol.

Current research on ACO traits has not utilized all possible classification methods. Therefore, such contributing and provoking factors as spirometry findings; patient record history; the prognosis of patient status, including disease exacerbating conditions; and intrinsic atopy-related markers should be considered in future clinical trials. 

Longitudinal studies are required to enable a deeper understanding of these phenotypes and how they influence management decisions. In addition, performing detailed omics research, including the respiratory microbiome, could considerably improve phenotyping.

## 9. Conclusions and Clinical Care Points

Asthma and COPD have characteristic symptoms, yet patients with both are prevalent. However, there is no globally accepted definition of ACO, and it may not be considered a distinct disease or clinical entity. Recognizing patients with both disorders helps guide clinical therapy. As with asthma and COPD, ACO patients are heterogeneous and presumably have multiple underlying disease processes.

Besides the age and smoking history of the patient, recurrent pulmonary infections and inflammation have been considered as provoking factors contributing to the worsening of pulmonary function in elders.

The examination of suspected ACO is similar to asthma and COPD, focusing on airflow limitation and BD reversibility. A chest radiograph can help rule out other causes of dyspnea besides asthma, COPD, and ACO. Laboratory findings that monitor elevated total serum IgE, eosinophilia, intrinsic or extrinsic atopic hypersensitivity, and efficient CT scan might be useful for clarifying mysterious diagnostic cases.

Although the diagnosis of ACO depends on a mix of clinical features, not all are required: age 40; non-reversible airflow limitation; improvement in FEV_1_ by 10% of the predicted value after BD; a history of asthma, atopy or allergies; and exposure to agents such as smoke. Eosinophil count >300 cells/µL supports ACO or asthma diagnosis.

No single biomarker can be used to identify and classify ACO phenotypes. However, the number of eosinophils in the blood and BD reversibility can aid in phenotyping ACO. While total IgE, allergen-specific IgE, FeNO, chest imaging, and metabolic markers may assist in diagnosing ACO, their role in phenotyping ACO is unclear.

Nonpharmacologic treatment options for ACO patients include smoking cessation, annual influenza and pneumococcus vaccinations, inhaler technique education, avoidance of provoking allergy factors for people susceptible to known allergens, and pulmonary rehabilitation.

Free and easy access to BDs inhalers should be available for any patient with ACO to alleviate the symptoms rapidly. Similar to asthma, the treatment of ACO tends to be escalated to counteract clinical manifestations and prevent further worsening. This may involve increasing ICS doses or adding LAMA, ICS, and LABA (triple therapy). However, despite triple inhaled treatment in patients with persistent symptoms or exacerbations, they should be assessed for manifestations that indicate a potential advantages from monoclonal based pharmacotherapy such as Omalizumab, mepolizumab, or benralizumab developed for asthma. When selecting biologic drugs for ACO patients, we utilize the same criteria for severe persistent asthma.

It is essential to conduct large-scale trials to evaluate clinically relevant outcomes in patients with well-defined ACO.

## Figures and Tables

**Table 1:** Clinical features of asthma, COPD, and ACO.

More Likely Asthma If:	More Likely COPD If:
Prior diagnosis of asthma	Previous diagnosis of COPD, (chronic bronchitis or emphysema)
Onset age < 20 years	Onset age > 40 years
Variation in respiratory symptoms within short periods	Persistence respiratory symptoms
Worsening of symptoms at night or early morning	Daily symptoms and exertional dyspnea
Symptoms triggered by exposure to allergens, dust, exercise, or emotions/laughter	Chronic cough and sputum precede onset of dyspnea, unrelated to triggers
Family history of asthma, atopy, or eczema	Heavy exposure to a risk factor (such as smoking and biomass fuel)
Documented airflow limitation variability (spirometry or peak flow meter)	Documented persistent airflow limitation (post-BD FEV_1_/FVC < 70%)
Normal lung function between symptoms	Abnormal lung function between symptoms
No worsening of symptoms over time (symptoms vary either seasonally or from year to year)	Symptoms slowly worsening and progressive over time (years)
May improve spontaneously or have an immediate response to BDs or ICS over weeks	Rapid-acting BD provides limited relief
Normal chest radiograph	Severe hyperinflation on chest radiograph

If there are ≥3 items present for either asthma or COPD, it is likely that the patient has that disease. A similar number of items for both asthma and COPD suggest the possibility of ACO. Adapted from [8].

**Table 2:** Proposed ACO diagnostic criteria according to previous ten years’ studies.

No.	Reference	Major Criteria	Minor Criteria
1.	[13]	COPD + positive BD test (increase in FEV_1_ ≥ 15% and ≥400 mL). COPD + sputa eosinophilia. COPD + asthma history.	COPD + elevated total serum IgE. COPD + atopy history. COPD + BD test (increase in FEV1 ≥ 12% and ≥ 200mL above baseline on ≥2 events).
	Required points	2	-
	OR	1	2
2.	[12]	COPD + positive BD test (increase in FEV1 ≥ 15% and ≥400 mL). COPD + positive Methacholine challenge test. COPD + FeNO ≥ 45–50 ppb and/or sputa eosinophils > 3%. COPD + asthma history.	COPD + BD test (increase in FEV1 > 12% and >200 mL). COPD + elevated IgE. COPD + atopy history.
	Required points	2	-
	OR	1	2
3.	[9]	COPD + asthma history. COPD + BD response to Albuterol >15% and 400 mL.	COPD + IgE > 100 IU. COPD + atopy history. COPD + 2 separated BD responses to Albuterol (>12% and 200 mL). COPD + blood eosinophils > 5%.
	Required points	1	-
	OR	-	2
4.	[10]	COPD + FEV1/FVC < 0.7 or LLN in patients ≥ 40 years of age. COPD + ≥10-pack-year of tobacco smoking OR equivalent air pollution exposure. COPD + documented asthma history before the age of 40 OR BD reversibility > 400 mL in FEV1.	COPD + documented atopy or allergic rhinitis history. COPD + BD reversibility of FEV1 ≥ 200 mL and 12% from baseline on ≥2 visits. COPD + peripheral blood eosinophil count of ≥300 cells/mL.
	Required points	3	1
		ACO in a COPD patient:	
5.	[11]	A large degree of time variation in airway obstruction: FEV1 variation ≥ 400 mL. A high degree of BD response: >200 mL and 12% above baseline.	Patient or family history of atopy and/or IgE sensitivity to ≥1 airborne allergen. Increased blood or sputum eosinophils or increased FeNO. Asthma diagnosed prior to the age of 40. Symptom variability. Age (in favor of asthma).
		ACO in an asthma patient:	
		Persistence over time of airflow obstruction (FEV1/FVC < 0.7 or <LLN). Exposure to noxious particles or gases with ≥10-pack-year smoking.	Non-response to BD tests. Decreased lung diffusion capacity. Few variabilities in airway obstruction. Age in favor of COPD (>40 years). Presence of emphysema on chest CT scan.
	Required points	2	1
6.	[14]	Age > 35 years. Post-BD FEV1/FVC < 70%. ≥10-pack-year tobacco smoke.	Current asthma diagnosis. No current asthma diagnosis but a BD response to Albuterol ≥ 15% and 400 mL and/or blood eosinophils ≥ 300 cells/µL.
	Required points	3	1

BD: bronchodilator; FEV_1_: forced expiratory volume in one second; FVC: forced vital capacity; COPD: chronic obstructive pulmonary disease; IgE: immunoglobulin E; FeNO: fraction of exhaled nitric oxide; ppb: parts per billion; ACO: asthma COPD overlap; IU: international units; LLN: lower limit of normal.

**Table 3:** The concluded proposed data collection for ACO diagnostic.

Field	Criteria	Comments
Demographic data	Age ≥ 40 years.	
Medical history	A documented asthmatic history before the age of 40.	
	Prior symptoms of asthma or allergic rhinitis.	
	History of atopy and/or allergies.	
	≥10-pack-year of tobacco smoking or equivalent air pollution.	
Symptoms	The frequency, intensity, and duration of respiratory symptoms.	Respiratory symptoms (e.g., dyspnea including exertional dyspnea, cough, sputum) are persistent, but variability in symptoms may be prominent.
	Exercise limitation.	
Spirometry	Post-BD FEV_1_/FVC < 70% or LLN and BD increase in FEV_1_ > 12% and 400 mL.	The assessment of ACO must involve spirometry before and after BD. These tests confirm airflow limitation (obstruction) and evaluate its reversibility. The airflow limitation is not completely reversible, however there has been historical variance. ACO patients usually have a BD response, defined as a post-BD rise in FEV_1_ or FVC of more than 10% of the predicted value; however, asthma patients usually have an increase in FEV_1_ or FVC of more than 15% of their predicted value. Post-BD FEV1 and FEV_1_/FVC are poor in ACO patients. Normal post-BD values suggest asthma.
	FEV_1_ < 80%.	
	Persistent partially reversible airflow obstruction (without normalization of obstruction).	While airflow limitation is required for ACO diagnosis, it does not assist distinguish ACO from asthma or COPD.
Laboratory tests	A high level of total serum IgE (>100 international units/mL).	Not routinely obtained. May point a practitioner to asthma or ACO.
	Elevated peripheral blood eosinophil count (>300 cells/µL).	May point a practitioner to asthma or ACO.
	Evidence of allergic disease.	Not routinely obtained. May point a practitioner to asthma or ACO.
	Elevated sputum eosinophil counts.	Not routinely obtained. More common in asthma or ACO than COPD.
	Alpha-1 antitrypsin deficiency.	Recommended for all patients with fixed-airflow limitation.
Imaging	A chest radiograph is often used to diagnose chronic symptoms or an exacerbation. The chest radiograph may indicate hyperinflation in ACO patients, but it does not distinguish between asthma, COPD, and ACO.	
	If there is diagnostic uncertainty, high-resolution computed tomography may be helpful. Small airway disease without emphysema may be ACO, although asthma (without COPD) and bronchiolitis obliterans should be evaluated. Severe emphysema is more common in COPD than ACO [8].	

Adapted from [3,4,7].

**Table 4:** The recommended non-pharmacological therapy for patients with ACO.

Intervention	Comments
Smoking cessation	For all ACO patients who smoke. Avoiding exposure to additional sources of smoke and irritating inhalants.
Vaccination	
Annual influenza vaccine	For all adults.
Pneumococcus vaccine	Decreases the rate of exacerbations and community-acquired pneumonia.
Allergen avoidance	
Inhaler technique	At each appointment, the inhaler technique should be reviewed.
Pulmonary rehabilitation	These programs are undeniably beneficial for COPD patients and could be helpful supplementary resources for teaching inhaler techniques and keeping the most active lifestyle possible.

**Table 5:** The use of biologic agents in patients with ACO features.

Class	Biologic Agents	Evidence	References
Anti-IgE therapy	Omalizumab	Reduces exacerbations and improves symptoms moderately in allergic asthma patients with elevated serum IgE levels and sensitivity to perennial allergens; may be advantageous in ACO.	[56,60,61,62,63,64,65]
Anti-IL-5/IL-5 receptor alpha (IL-5Ra) therapies	Benralizumab Mepolizumab Reslizumab	Minimizes asthma exacerbations, improves asthma symptoms and quality of life, and reduces systemic corticosteroid use.	[66,67,68]
IL-4 receptor alpha (IL-4Ra) and IL-13 therapies	Dupilumab	Enhances lung function and decreases exacerbations in severe asthma, with patients with higher blood eosinophil levels benefiting the most. However, efficacy in COPD and ACO is not established.	[59,69]
Anti-thymic stromal lymphopoietin-IgG2λ	Tezepelumab	Add-on controller treatment of severe asthma in adults and children ≥ 12 years of age.	[70,71]

## Data Availability

Not applicable.
